# p16INK4a inhibits the proliferation of osteosarcoma cells through regulating the miR-146b-5p/TRAF6 pathway

**DOI:** 10.1042/BSR20181268

**Published:** 2019-02-01

**Authors:** Mingwei Jiang, Wenjia Lu, Xiaomin Ding, Xiaodong Liu, Zhen Guo, Xu Wu

**Affiliations:** 1 Department of Orthopedics, Yangpu Hospital, Tongji University School of Medicine, Shanghai 200090, P.R. China; 2 Department of Orthopedics, Xinhua People’s Hospital, Xinhua City, Jiangsu Province 225700, P.R. China

**Keywords:** miR-146b-5p, Osteosarcoma Cells, p16INK4a, tumor suppressors

## Abstract

Down-regulation of p16INK4a and miR-146b-5p contributes to tumorigenesis in osteosarcoma (OS). However, the correlation between p16INK4a and miR-146b-5p in OS proliferation remains largely unknown. In the present study, we demonstrated that miR-146b-5p expression was positively correlated with p16INK4a in OS, but inversely correlated with TNF receptor associated factor 6 (TRAF6) expression. Overexpression of miR-146b-5p dramatically suppressed OS cell proliferation. Mechanistically, we validated TRAF6 as a direct functional target of miR-146b-5p and found that miR-146b-5p overexpression significantly decreased the level of phosphorylated PI3k and Akt, which are the pivotal downstream effectors of TRAF6. Moreover, TRAF6 expression was positively correlated with Ki-67 but inversely correlated with miR-146b-5p expression. In OS cells, silencing of TRAF6 mimicked the anti-tumor effects of miR-146b-5p. p16INK4a is an important tumor suppressor gene frequently down-regulated in OS. We found that this inhibitory effect is associated with the suppression of the miR-146b-5p, and is mediated via up-regulating TRAF6 expression. Our findings identified p16INK4a and miR-146b-5p as tumor suppressors, and suggested p16INK4a, miR-146b-5p and TRAF6 as potential therapeutic candidates for malignant OS.

## Introduction

Osteosarcoma (OS) is a high-grade malignant cancer of skeleton, which originates from immature osteoid matrix deposition by spindle cells originated from mesenchymal tissue [1,2]. OS primarily affects long bones, with potential spread to other bones [3]. OS is the most prevalent bone cancer in children and adolescents [4]. However, it is bimodally distributed among different age groups of 10–14 years and 60 years [4].

Surgery, radiotherapy and chemotherapy are primary treatment modalities for OS. Several clinical and preclinical agents are also investigated for the targeted therapy against OS, including denosumab, glembatumumab vedotin, etc [5]. However, the therapeutic outcome of these strategies is still suboptimal and there is an urgent need for new and effective therapies against OS. One class of emerging therapeutic molecules against cancers is microRNA (miRNA). MiRNAs are small non-coding regulatory molecules of RNA having 15–25 nucleotides [6]. MiRNAs perform a broad spectrum of biological functions by binding with 3′UTR of genes [7,8]. They are also involved in the other biological processes, such as apoptosis, metabolism, development, proliferation and cellular differentiation [8]. They are also known to regulate different biological processes like cancer, stemness and immunity [8]. Studies have also shown that the genetic regions altered in human cancers express almost 50% of discovered miRNA genes, suggesting their direct role in tumorigenesis [9].

Gao et al. [10] first identified 182 miRNAs expression in the human OS cell lines, indicating an important role of miRNA in OS pathogenesis. Another study on OS tumors identified 38 unique miRNAs expressed in tumors as compared with normal osteoblasts, among which 28 miRNAs were down-regulated in the cancerous tissues while 10 miRNAs were up-regulated [11]. Further studies also identified different miRNAs specific to OS, including miR-150, miR-652, miR542-5p and miR132b [12]. Characterization of these identified miRNAs in the OS may give insight into their use as biomarker or as therapeutic agents [7].

The tumor suppressor gene p16INK4a (cyclin-dependent kinase inhibitor p16) and miR-146b-5p have been previously found to be linked to a number of human malignancies including cancers. For example, in breast cancer, down-regulation of p16 results in breast stromal fibroblasts activation [13]. The p16 down-regulation was also shown to result in IL6 repression, which was mediated through miR-146b-5p, as p16 positively regulates miR-146b-5p [14]. miR-146b-5p represses various carcinogenic pathways, such as epithelial-to-mesenchymal transition, migration and invasion [15–17], and in the metastasis of breast cancer [18].

In OS, miR-146b-5p overexpression was shown to contribute to tumorigenesis by promoting tumor migration and invasiveness [19]. Overexpression of p16^INK4a^ increases the patient survival in OS [20]. However, the underlying mechanism of p16INK4a and miR-146b-5p in cancer remains largely unknown.

Given the significant role of miR-146b-5p and p16INK4a in cancer, and the potential of these molecules as new therapeutic agents for OS, herein we aim to investigate the possible correlation of p16INK4a and miR-146b-5p in OS and elucidate the precise molecular mechanism. Studies have found that TNF receptor associated factor 6 (TRAF6) induces p16INK4a production [21]. Proliferation of OS is governed by the expression of p16INK4a, miR-146b-5p and TRAF6. These data provide useful information for the clinical utility of p16INK4a in OS to potentially improve the therapeutic outcome of the disease.

## Method

### Cell culture

HFSN1 (primary normal human skin fibroblast), U2OS (human OS cell line, which does not express p16 due to promoter hypermethylation) and EH1 (derived from U2OS, which expresses p16) were gifts from American Type Culture Collection. SNB19 and U251 were acquired from the China Academia Sinica Cell Repository (Shanghai). All cells were cultured at 37°C and 5% CO_2_ in DMEM/F12 medium supplemented with 10% FBS and 1%antibiotic/antimycotics.

### Cell migration and invasion assay

The migration and invasion of cells were measured using wound healing assay and transwell assay, respectively. Briefly, for wound healing assay, cells were seeded in six-well plates and cultured to 90% confluence. After medium removal, a wound gap was enforced on the monolayer cells that were using a sterile pipette tip. At 24 h after incubation, the width of the wounding gaps was measured and the migration rate was calculated as follows: migration rate = migration distance/original distance. For transwell assay, cells (5 × 10^4^ per well) were suspended in 200 ml serum free medium in the upper chamber of the transwell apparatus (8 mm, BD Biosciences), which was coated with BD BioCoat Matrigel. After incubation, the cells on the upper membrane surface were removed with a cotton tip. Then, membrane was then fixed and stained by violet crystalline.

### Western blot assay

The total protein was extracted using the RIPA buffer (Sigma–Aldrich, St. Louis, MO) supplemented with protease inhibitors cocktail (Roche, Diagnostics, Mannheim, Germany). After protein concentration measurement using the BCA assay, equal amounts of protein were separated by SDS–PAGE (sodium dodecyl sulfate-polyacrylamide gel electrophoresis), followed by transferring to PVDF membrane (Millipore, Bedford, MA). Non-fat milk was used to block the membranes. Corresponding primary antibodies and secondary antibodies were then sequentially added, with extensive washing with TBST in between. Protein bands were detected using ECL detection kit (Beyotime Biotechnology, Shanghai, China).

### Locked nucleic acid-modified oligonucleotide probes

Locked nucleic acid (LNA)-modified oligonucleotide probes with digoxin at their 5′ ends were synthesized by TaKaRa. The sequences of miR-146b-5p probe and scramble control probe were 5′-ALgCCTLaTGGLaATTLcAGTLtCTCA-3′ and 5′-CLgTAT LaGGCLcCAALgAATLtAGG-3′, respectively. La, Lt, Lc and Lg were LNA monomers corresponding to the bases A, T, C and G.

### Luciferase plasmid construction

The prediction of the candidate target genes of miR-146b-5p were performed using miRTarBase (http://mirtarbase.mbc.nctu.edu.tw/) and TargetScan (http://www.Targetscan.org/). The pEZX-MT01 Luciferase miRNA Expression Reporter Vector (GeneCopoeia) was applied to construct the wild (TRAF6-WT) and mutant (TRAF6-mut) reporter vectors of TRAF6 3′-untranslated region (3′-UTR). The coding-sequences of predicted mR-146b-5p binding site (1 and 2, or 3 and 4) were deleted from TRAF6 3′-UTR cDNA, which were used to construct p-MT1 or p-MT2 by site-directed mutagenesis. The sequences of the inserts in three vectors were validated by DNA sequencing.

### Dual-luciferase reporter assay

HEK293T cells, which were seeded and cultured in 96-well plates, were co-transfected with 0.15 μg TRAF6-WT, TRAF6-Mut and 0.08 μg miR-146b-5p mimics or scrambled sequence (negative control) using X-tremeGENE siRNA Transfection Reagent (Roche). The mock controls were constructed by transfecting the cells using the aformentioned recombinant reporter vectors. The activities of renilla and firefly luciferases were detected with Dual-Luciferase Reporter Assay System (Promega) on a Synergy 2 Microplate Reader Fluorometer (BioTek). The results were normalized to the activity of firefly luciferase.

### miR-146b-5p mimics and TRAF6 siRNA transfection

The dsRNA oligonucleotides of miR-146b-5p mimics, small interfering RNA silencing TRAF6 (TRAF6 siRNA) and scrambled sequence (negative control) were purchased from GenePharma. The U87MG, SNB19 and U251 cells of miR-146b-5p group, TRAF6 siRNA group and negative control group were transfected with the corresponding dsRNA oligonucleotides using X-tremeGENE siRNA Transfection Reagent (Roche). The mock groups were treated with the transfection reagent of the same volume.

### Quantitative RT-PCR (qRT-PCR)

Total RNA was extracted using TRIzol reagent (Invitrogen), followed by quantification with Stem-loop qRT-PCR Detection Kit (GenePharma). GoTaq qPCR Master Mix Kit (Promega) and a Reverse Transcription System were used for the quantitative RT-PCR (qRT-PCR) detection of TRAF6 mRNA. U6 and glyceraldehyde-3-phosphate dehydrogenase (GAPDH) were used as the internal controls. All reactions were performed on a CFX Connect™ Real-Time PCR Detection System (Bio-Rad). The fold changes of miR-146b-5p and TRAF6 mRNA levels were calculated by the 2^−ΔΔ*C*^_t_ method.

### Tumor cell proliferation assay (CCK8 assay)

Cells were seeded and cultured in 96-well plates. At 1, 2, 3, 4, 5 days after transfecting miR-146b-5p mimics, TRAF6 siRNA or scrambled siRNA (negative control), 20 μl of Cell Counting Kit-8 (Beyotime) was added to each well and incubated for 2 h at 37°C. The absorbance at 450 nm was measured on a Synergy 2 microplate reader (BioTek).

### Statistical analyses

All statistical analyses were performed with SPSS 18.0 software. Data were presented as the mean ± standard deviation (SD). The differences among/between sample groups were analyzed by one-way ANOVA or Student’s *t* test. Statistical significance was assigned at *P*<0.05 (*). All the experiments of cell lines were performed at least three times with triplicate samples

## Result

### p16INK4a regulates the expression of miR-146b-5p and TRAF6

**Figure 1:**
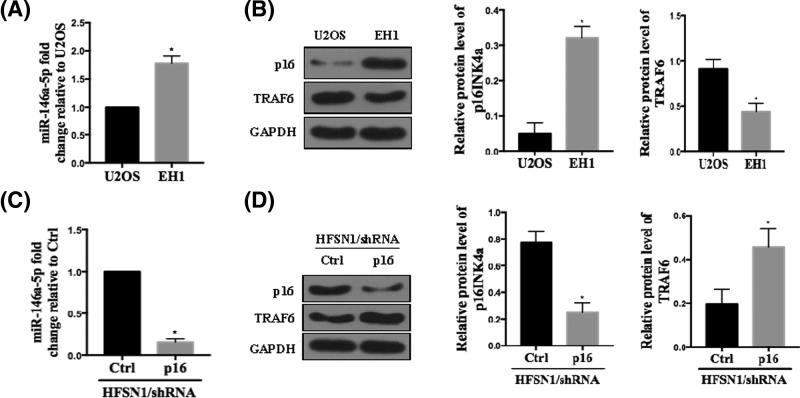
p16INK4a regulates the expression of miR-146b-5p and TRAF6 (**A**) Total RNA was extracted from the indicated cells, and mature miR-146b-5p was amplified by qRT-PCR. The values were normalized against U6 and calculated as fold change relative to control U2OS cells. Histogram shows mean ± SD of three independent experiments. (**B**) Western blot assay examined the p16 and TRAF6 expression in the indicated cells. The relative expression level of p16 and TRAF6 was normalized against GAPDH. (**C**) Total RNA was extracted from the HFSN1 cells transfected with shRNA-vector or shRNA-p16, and mature miR-21-5p was amplified by qRT-PCR. The values were normalized against U6 and calculated as fold change relative to control cells. (**D**) Western blot assay examined the p16 and TRAF6 expression in the indicated cells. The relative expression level of p16 and TRAF6 was normalized against GAPDH. All experiments were performed at least in triplicate and the data are presented as the mean ± SD. *P<0.05; *n* = 5 or 6.

As shown in Figure 1A, qRT-PCR analysis indicated that the level of mature miR-146b-5p was 2-fold higher in p16-expressing EH1 cells than that of p16-deficient U2OS cells. This evidence suggest that p16 promotes the expression of miR-146b-5p. To further confirm the positive correlation of p16 with miR-146b-5p, we investigated the effect of p16 shRNA transfection on miR-146b-5p expression in normal human skin fibroblast (HFSN1). Figure 1C shows that p16 down-regulation with specific shRNA halved p16 level and induced a 5-fold reduction of the levels of miR-146b-3p, as compared with control cells transfected with a scrambled shRNA. Conversely, Western blot assay showed that the level of TRAF6 was up-regulated in p16-deficient cells (Figure 1B,D).

### TRAF6 is a direct target of miR-146b-5p

**Figure 2:**
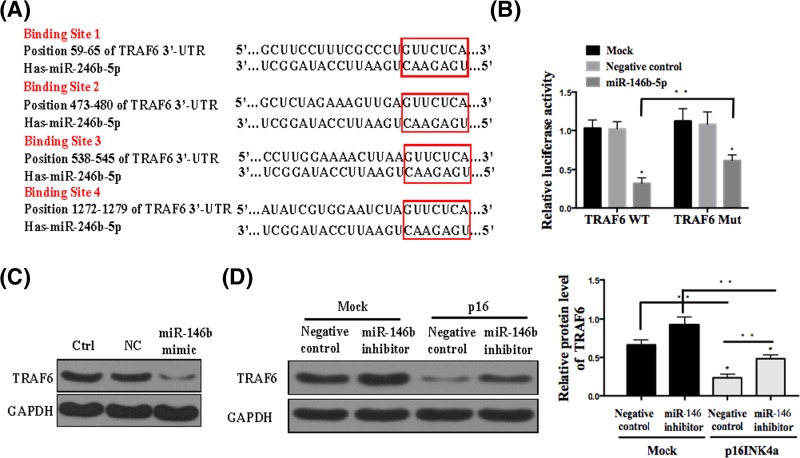
TRAF6 is a direct target of miR-146b-5p (**A**) Four miR-146b-5p binding sites in TRAF6 3′-UTR predicted with TargetScan. Wild (TRAF6-3′-UTR-WT) and mutant (TRAF6-3′-UTR-mut) TRAF6 3′-UTRs carried in recombinant luciferase mRNAs transcribed by TRAF6 WT and TRAF6-mut. (**B**) Luciferase reporter assays in HEK293T cells transfected with TRAF6 WT and TRAF6-mut (Mock), and co-transfected with TRAF6 WT, TRAF6-mut and scrambled sequence (negative control) or miR-146b-5p mimics. (**C**) Western blot assay analyses of TRAF6 expression in the EH1 cells transfected with miR-146b-5p mimics. (**D**) Western blot assay analyses of TRAF6 expression in the EH1 cells transfected with miR-146b-5p inhibitor followed with or without p16 treatment. The relative expression level of TRAF6 was normalized against GAPDH. All experiments were performed at least in triplicate and the data are presented as the mean ± SD. *P<0.05; **P<0.05; *n* = 5 or 6.

TargetScan and miRTarBase predictions revealed that the 3′-UTR of TRAF6 mRNA encompassed four conserved miR-146b-5p binding sites (Figure 2A). To confirm the prediction results, we constructed two recombinant luciferase reporter vectors of TRAF6 3′-UTR (TRAF6 WT and TRAF6 mut). The recombinant luciferase mRNA transcribed by TRAF6 WT carried all miR-146b-5p binding sites predicted in TRAF6 3′-UTR, while the one transcribed by TRAF6 mut lacked all the predicted binding sites (Figure 2A). The dual-luciferase assay showed that miR-146b-5p could effectively suppress the luciferase activity delivered by the recombinant reporter vectors in HEK 293T cells (Figure 2B). To further verify whether miR-146b-5p directly induces TRAF6 knockdown, we monitored the changes of miR-146b-5p and TRAF6 levels in the EH1 cell lines transfected with miR-146b-5p by Western blotting. As shown in Figure 2C, TRAF6 was significantly decreased, as compared with negative control. The EH1 cell line was transfected with miR-146b-5p inhibitor, with or without recombinant p16 treatment. As shown in Figure 2D, miR-146b-3p inhibitor repressed TRAF6 expression, while p16 treatment further reduced the level of TRAF6.

### miR-146b-5p and TRAF6 involve OS progression *in vitro*

**Figure 3:**
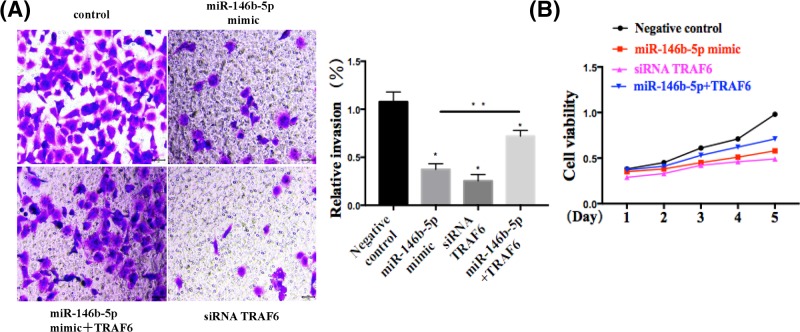
miR-146b-5p and TRAF6 involves OS progression *in vitro* (**A**) miR-146b-5p affects OS cells migration by wound healing assay in EH1 cells. In miR-146b-5p group, cells transfected with miR-146b-5p mimics. TRAF6 siRNA group, cells transfected with TRAF6 siRNA. TRAF6 group, cells co-transfected with miR-146b-5p mimics and pcDNA/TRAF6. (**B**) miR-146b-5p affects OS cells proliferation by CCK8 assay in EH1 cells. *P<0.05 vs. control group, **P<0.05 vs. miR-146b-5p group, *n* = 5 or 6.

Next, we determined the effect of miR-146b-5p and TRAF6 on the tumor growth *in vitro*. EH1 cells were co-transfected miR-146b-5p mimic with or without TRAF6 siRNA. Consistently, miR-146b-3p mimics evidently suppressed OS cell migration and invasion. Moreover, pcDNA-TRAF6 rescued the inhibition of miR-146b-5p on cell invasion and migration (Figure 3A,B). In conclusion, these results suggested that miR-146b-3p might participate in OS progression in part by repressing TRAF6 expression.

### miR-146b-5p/TRAF6 inhibits the p16-mediated proliferation of OS cells

**Figure 4:**
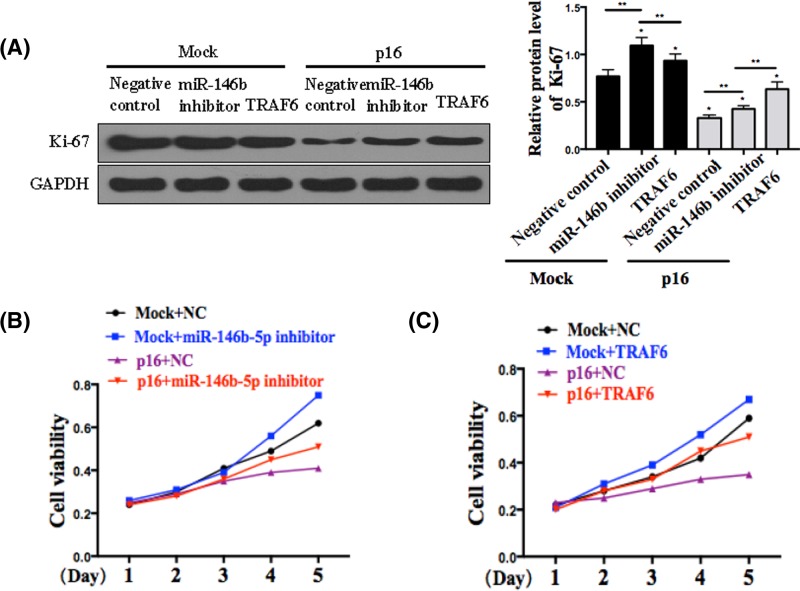
miR-146b-5p/TRAF6 inhibits the p16-mediated proliferation of OS cells (**A**) Western blot analysis of Ki-67 expression in EH1 cells transfected with miR-146b-5p inhibitor or pcDNA-TRAF6 followed with or without p16 treatment. The relative expression level of Ki-67 was normalized against GAPDH. (**B**) and (**C**) Growth curves from the above transfected cells assessed by CCK8 assay. All experiments were performed at least in triplicate and the data are presented as the mean ± SD. *P<0.05; **P<0.05; *n* = 5 or 6.

To clarify the associations of cell proliferation with the expressions of miR-146b-5p and TRAF6 in OS, we analyzed the expression of proliferation marker, Ki-67, using Western blot assay. We found that transfection with miR-146b-5p inhibitor and TRAF6 uniformly significantly reduced Ki-67 expression, and OS cell proliferation, as measured by Western blotting (Figure 4A) and CCK8 proliferation assay (Figure 4B), respectively. Moreover, treatment with p16 shRNA inhibited the proliferation of OS cells.

### P16/miR-146b-5p affects PI3K/Akt pathway in OS cells

**Figure 5:**
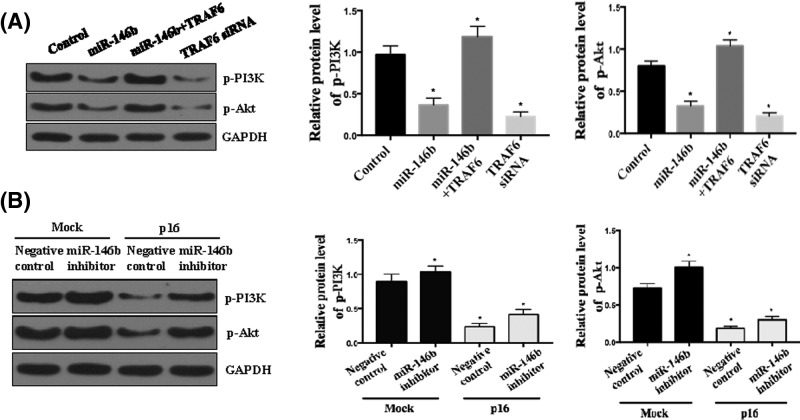
P16/miR-146b-5p affects PI3K/Akt pathway in OS cells (**A**) MiR-146b-5p affects PI3k/Akt pathway in OS cells. EH1 cell transfected with miR-146b-5p mimics, TRAF6 siRNA or co-transfected with miR-146b-5p mimics and p-PI3k and p-Akt detected by Western blotting. The relative expression levels of p-PI3k and p-Akt were normalized against GAPDH. (**B**) p16/miR-146b-5p affects PI3k/Akt pathway in OS cells. EH1 cell transfected with miR-146b-5p inhibitor followed with or without p16 treatment, and p-PI3k and p-Akt detected by Western blotting. The relative expression levels of p-PI3k and p-Akt were normalized against GAPDH. All experiments were performed at least in triplicate and the data are presented as the mean ± SD. *P<0.05; *n* = 5 or 6.

PI3k/Akt pathway plays a central role in growth, proliferation and cell survival [22]. Previous work showed that TRAF6 mediated PI3k/Akt activation by phosphorylation [22]. Therefore, we speculated that p16/miR-146b-5p regulate OS through PI3k/Akt activation. As shown in Figure 5A, miR-146b-5p evidently decreased pAkt, and p-PI3k expression in OS cells, which was reversed by TRAF6 overexpression, indicating that miR-146b-5p inhibited TRAF6-induced PI3k/Akt activation. Further, EH1 cells were transfected with miR-146b-5p inhibitor with or without p16 shRNA treatment, miR-146b-5p inhibitor increased pAkt and p-PI3k expression in OS cells, while p16 shRNA evidently decreased pAkt and p-PI3k expression (Figure 5B).

## Discussion

In the present study, we identified that p16INK4a and miR-146b-5p are suppressors of OS. A strong correlation between p16INK4a and miR-146b-5p expression was observed. This is demonstrated by qRT-PCR analysis of p16 expressing cells (EH1) and p16-deficient cells (U2OS) (Figure 1), which clearly showed elevated expression of mature miR-146b-5p in EH1 cells as compared with U2OS cells. Previously, Al-Khalaf et al. [14] also showed positive correlation between p16 and miR-146b-5p expression at translational level. Our findings are in line with previous findings that p16INK4a and miR-146b-5p are tumor suppressors as miR-146b-5p is involved in the initiation of p16-mediated apoptosis [14]. Another study on breast stromal fibroblasts also showed positive relationship between these two, where they showed that p16 is involved in tumor suppression mediated by miR-146b-5p [13].

As a mechanistic study, we showed that TRAF6 is a target of miR-146b-5p. This was validated by predicting four binding sites of miR-146b-5p within TRAF6 sequence by TargetScan and miRTarBase, which were further confirmed by dual-luciferase assay. Moreover, Western blot analysis showed that TRAF6 expression decreased in the presence of miR-146b-5p and p16 (Figure 2). Indeed, TRAF6 is a putative oncogene in a variety of cancers including lung cancer [23], bladder cancer [24], breast cancer [25], prostate cancer [26] and skin cancer [27]. TRAF6 is also regulated by several cancer-related miRNAs, particularly miR-146a, which negatively regulates TRAF6 expression. TRAF6 3′UTR also bears multiple sites for miR-146 binding [28,29].

Consistently, we demonstrated that OS proliferation is correlated with miR-146b-5p and TRAF6 expression levels. Figure 3 clearly depicts the effects of both TRAF6 and miR-146b-5p on cell proliferation. In this experiment, the effect of miR-146b-5p on the tumor growth was determined by co-transfecting EH1 cells with miR-146b-5p mimic with or without TRAF6 siRNA. The results clearly showed negative effects of miR-146b-5p on cell migration and evasion. Together, these results suggest that OS progression is partly regulated by TRAF6 repression, which was mediated by miR-146b-5p. To corroborate this, Ki-67 expression was measured by Western blotting in tumor cells. Basically, Ki-67 is a cell proliferation antigen and its constitutive expression has been found in the dividing cells of mammals [30]. Due to its continuous expression in proliferating cells Ki-67 DNA-binding protein have been widely used as proliferation marker in tumor grading [31]. Ki-67 was analyzed following miR-146b-5p inhibition or TRAF6 overexpression to determine the cell viability in OS by Western blot assay and CCK8 proliferation assay. It can be inferred from this experiment that both TRAF6 and miR146b-5p inhibitor reduced the OS proliferation as Ki-67 expression decreased. Further, it was revealed that proliferation of OS cell is inhibited by p16 treatment. Concomitantly, p16 was found to be prominently correlated with this regulation of OS proliferation.

Dysregulation of P13K/Akt pathway is a putative event in OS. In fact, this pathway governs multiple pathological and physiological processes [22], including several types of cancer [32], through the regulation of cell proliferation, growth and survival [33]. Advanced stage cancer patients of OS are also characterized by mutations or alterations in this pathway, suggesting that its dysregulation is an essential feature for metastatic OS [34]. We also found that miR-146b-5p and TRAF6 play an important role in the regulation of PI3K/Akt signaling pathway. In OS cells, p-P13K and pAkt expression has been shown to be decreased by miR-146b-5p, which was reversed by introducing TRAF6. This evidence suggest that inhibition of TRAF6 induced PI3k/Akt activation via miR-146b-5p (Figure 5A). Further, introduction of miR-146b-5p inhibitor in the presence or absence of P16 led to the findings that p16 decreases the expression of pAkt and p-PI3k, while pAkt and p-PI3k expression increases in the absence of miR-146b-5p (Figure 5B).

The present study suggests the potential use of miR-146b-5p as therapeutics in OS. Existing treatments for OS suffer from high invasiveness, severe side effects and suboptimal clinical outcome. Surgery, such as amputation or more recently by limb salvage, is the primary treatment option to stop metastasis of cancer. In limb salvage, all cancerous tissue is carefully removed from the limb, leaving its function preserved. Limb preservation is known as the safest method as compared with amputation [35]. However, these procedures are highly invasive, which inevitably lead to poor quality of life. In recent days, chemotherapy followed by surgery has significantly improved OS treatment [3]. However, chemotherapy is also associated with several side effects, including cardiomyopathy induced by Adriamycin [36]. Although these methods increase the life span of patient but still have limitations [37]. Targeted cancer therapeutics, such as denosumab, a monoclonal antibody against receptor activator of nuclear factor κB ligand (RANKL) has been developed and tested clinically. It disrupts RANK signaling required for motility and growth for cancerous cells. Preclinical studies have shown the potential of different microRNAs as therapeutic agents against various cancers. These miRNAs include miR-34, miR-155, miR-15/16 etc [38]. Our study also clearly demonstrated that miR-146b-5p suppresses the migration and invasion of OS cells *in vitro* by altering the PI3k/Akt pathway, which is responsible for cell growth and proliferation. Therefore, it can be concluded that miR-146b-5p may have therapeutic potential against OS. However, more studies are needed to validate this hypothesis.

## Availability of data and materials

The analyzed data sets generated during the study are available from the corresponding author on reasonable request. All authors have read and approved the final manuscript.

## Abbreviations

OS: osteosarcoma
TRAF6: TNF receptor associated factor 6
